# Supplementary material for: Associations between Single Nucleotide Polymorphisms from the Genes of Chemokines and the CXCR2 Chemokine Receptor and an Increased Risk of Endometrial Cancer
Source: Cancers (Basel). 2023 Nov 14;15(22):5416. doi: 10.3390/cancers15225416 (PMC10670474; doi:10.3390/cancers15225416)
Supplement: Supplementary file 1 [file cancers-15-05416-s001.zip › Supplementary Tables. Associations between single nucleotide polymorphisms from genes of chemokines (...). rev.pdf]

TITLE PAGE

Title: Associations between single nucleotide polymorphisms from the genes of chemokines and the CXCR2 chemokine receptor and an increased risk of endometrial cancer

Funding: This research was funded by the Polish Ministry of Science & Higher Education, Polish Mother's Memorial Hospital – Research Institute (the funds supporting statutory research projects).

Author List and Affiliations:

Wioletta I. Wujcicka [1*], Agnieszka Zając [2], Krzysztof Szyłło [2, 3], Hanna Romanowicz [4], Beata Smolarz [5], Grzegorz Stachowiak [2]

[1] Scientific Laboratory of the Center of Medical Laboratory Diagnostics and Screening, Polish Mother's Memorial Hospital - Research Institute, Lodz, 93-338, Poland

[2] Department of Operative Gynecology and Gynecologic Oncology, Polish Mother's Memorial Hospital - Research Institute, Lodz, 93-338, Poland

[3] Department of Operative and Endoscopic Gynecology, Medical University of Lodz, Lodz, 93-338, Poland

[4] Department of Clinical Pathomorphology, Polish Mother's Memorial Hospital - Research Institute, Lodz, 93-338, Poland

[5] Laboratory of Cancer Genetics of the Department of Clinical Pathomorphology, Polish Mother's Memorial Hospital - Research Institute, Lodz, 93-338, Poland

Corresponding Author:

* Wioletta I. Wujcicka, Ph.D., Scientific Laboratory of the Center of Medical Laboratory Diagnostics and Screening, Polish Mother's Memorial Hospital - Research Institute, Lodz, Poland, 281/289 Rzgowska St., 93-338 Lodz, Poland. Tel: +48 422711520, Fax: +48 422711510, e-mail: wioletta.wujcicka@iczmp.edu.pl, wwujcicka@yahoo.com

Supplementary Tables

Table S1A. Densitometric analysis of the PCR-RFLP products obtained for *CCL2* rs4586 genotyping, shown in Figure S1A.

	Band No. [a]	Relative Front [b]	Volume [c] (Intensity)	Band % [d]	Lane % [e]
Lane 1	1	0,716	13739320	100,00	36,62
Lane 2	1	0,784	1791108	68,64	8,34
	2	0,805	818136	31,36	3,81
Lane 3	1	0,784	6760044	42,36	16,63
	2	0,807	5380596	33,72	13,24
	3	0,854	3816360	23,92	9,39
Lane 4	1	0,780	7033534	65,30	14,20
	2	0,802	3737984	34,70	7,55
Lane 5	1	0,778	3749118	54,41	12,38
	2	0,802	3141574	45,59	10,38
Lane 6	1	0,776	4780178	78,70	16,73
	2	0,802	1294075	21,30	4,53
Lane 7	1	0,778	4970760	63,24	12,63
	2	0,800	2888800	36,76	7,34
Lane 8	1	0,776	1641600	39,56	6,61
	2	0,798	2507976	60,44	10,10
Lane 9	1	0,774	2232543	43,66	7,50
	2	0,798	2881153	56,34	9,68
Lane 10	1	0,704	7821958	100,00	17,02
Lane 11	1	0,778	4802004	44,02	11,69
	2	0,798	6106572	55,98	14,87
Lane 12	1	0,800	13146003	73,32	24,32
	2	0,846	4784180	26,68	8,85
Lane 13	1	0,778	8624628	49,38	15,00
	2	0,800	4265856	24,42	7,42
	3	0,846	4576932	26,20	7,96
Lane 14	1	0,704	10359760	100,00	23,49
Lane 15	1	0,776	2058444	72,98	7,27
	2	0,798	762156	27,02	2,69
Lane 16	1	0,702	8766520	100,00	25,31
Lane 17	1	0,309	6498258	6,55	5,97
	2	0,348	12331293	12,44	11,33
	3	0,383	6985602	7,04	6,42
	4	0,401	5057091	5,10	4,65
	5	0,426	4771533	4,81	4,38
	6	0,453	4089501	4,12	3,76

	7	0,484	3574350	3,60	3,28
	8	0,521	8125650	8,19	7,47
	9	0,539	4454034	4,49	4,09
	10	0,564	4549467	4,59	4,18
	11	0,586	3798561	3,83	3,49
	12	0,615	3113643	3,14	2,86
	13	0,646	5169840	5,21	4,75
	14	0,681	9336639	9,42	8,58
	15	0,720	6382194	6,44	5,86
	16	0,765	4390464	4,43	4,03
	17	0,819	6531798	6,59	6,00
Lane 18	1	0,767	6117150	65,60	14,46
	2	0,790	3207867	34,40	7,58
Lane 19	1	0,765	4809312	56,12	11,30
	2	0,790	3760632	43,88	8,84
Lane 20	1	0,763	3056472	51,49	9,53
	2	0,784	2880108	48,51	8,98
Lane 21	1	0,759	3922848	70,71	18,12
	2	0,782	1624572	29,29	7,50
Lane 22	1	0,757	8984628	49,94	22,21
	2	0,782	3375288	18,76	8,34
	3	0,833	5630796	31,30	13,92
Lane 23	1	0,753	4963248	59,88	14,38
	2	0,778	3325356	40,12	9,64
Lane 24	1	0,749	6609132	78,51	27,61
	2	0,772	1809180	21,49	7,56
Lane 25	1	0,747	6052212	28,62	13,49
	2	0,767	6429924	30,40	14,33
	3	0,821	8667720	40,98	19,32

[a] Band No., band number; [b] Relative Front, the relative movement of a band from top to bottom; [c] Volume, the sum of all intensities within the band; [d] Band %, percentage of a band volume compared to the volume of all bands in the lane; [e] Lane %, percentage of the band volume compared to the entire lane volume.

Table S1B. Densitometric analysis of the PCR-RFLP products obtained for *CCL5* rs2107538 genotyping, shown in Figure S1B.

	Band No. [a]	Relative Front [b]	Volume [c] (Intensity)	Band % [d]	Lane % [e]
Lane 1	1	0,709	10542360	100,00	34,60
Lane 2	1	0,728	3093760	100,00	12,90
Lane 3	1	0,728	2111240	100,00	9,04
Lane 4	1	0,709	433010	30,89	1,84
	2	0,728	968696	69,11	4,11
Lane 5	1	0,726	8792400	100,00	23,42
Lane 6	1	0,707	12011278	74,25	28,09
	2	0,726	4165395	25,75	9,74
Lane 7	1	0,724	4474120	100,00	14,05
Lane 8	1	0,722	9115914	100,00	23,22
Lane 9	1	0,720	9266250	100,00	21,97
Lane 10	1	0,699	5912672	74,82	13,50
	2	0,720	1990083	25,18	4,54
Lane 11	1	0,719	12905730	100,00	25,54
Lane 12	1	0,717	4097663	100,00	10,68
Lane 13	1	0,717	15455115	100,00	24,36
Lane 14	1	0,693	225034	23,73	0,73
	2	0,717	723424	76,27	2,34
Lane 15	1	0,693	4876404	63,15	11,94
	2	0,713	2845479	36,85	6,97
Lane 16	1	0,713	10029256	100,00	21,56
Lane 17	1	0,711	15163735	100,00	24,62
Lane 18	1	0,311	6738228	6,34	5,55
	2	0,350	12627540	11,88	10,40
	3	0,388	6759900	6,36	5,57
	4	0,406	5053032	4,75	4,16
	5	0,431	4852872	4,57	4,00
	6	0,459	4140036	3,89	3,41
	7	0,490	3607884	3,39	2,97
	8	0,528	8634240	8,12	7,11
	9	0,547	4746708	4,47	3,91
	10	0,573	4639500	4,36	3,82
	11	0,596	3891996	3,66	3,21
	12	0,626	3425040	3,22	2,82
	13	0,659	6187752	5,82	5,10
	14	0,695	11026476	10,37	9,08
	15	0,738	6906708	6,50	5,69
	16	0,785	4791024	4,51	3,95
	17	0,844	8272044	7,78	6,81

Lane 19	1	0,707	13368485	100,00	25,32
Lane 20	1	0,685	8757887	70,53	19,34
	2	0,705	3659455	29,47	8,08
Lane 21	1	0,701	6444956	100,00	17,34
Lane 22	1	0,697	13874960	100,00	36,91
Lane 23	1	0,675	8897814	68,27	20,52
	2	0,693	4136186	31,73	9,54
Lane 24	1	0,673	9897480	63,45	23,29
	2	0,693	5700722	36,55	13,42
Lane 25	1	0,671	9505920	68,26	22,98
	2	0,689	4420480	31,74	10,69
Lane 26	1	0,689	4304600	100,00	16,66

[a] Band No., band number; [b] Relative Front, the relative movement of a band from top to bottom; [c] Volume, the sum of all intensities within the band; [d] Band %, percentage of a band volume compared to the volume of all bands in the lane; [e] Lane %, percentage of the band volume compared to the entire lane volume.

Table S1C. Densitometric analysis of the PCR-RFLP products obtained for *CCL5* rs2280789 genotyping, shown in Figure S1C.

	Band No. [a]	Relative Front [b]	Volume [c] (Intensity)	Band % [d]	Lane % [e]
Lane 1	1	0,751	17956893	100,00	40,30
Lane 2	1	0,748	533448	100,00	3,13
Lane 3	1	0,751	22906080	100,00	48,65
Lane 4	1	0,751	12647340	93,29	37,62
	2	0,833	596412	4,40	1,77
	3	0,857	312912	2,31	0,93
Lane 5	1	0,751	12337802	100,00	29,40
Lane 6	1	0,751	9625160	100,00	22,88
Lane 7	1	0,753	4867609	100,00	14,73
Lane 8	1	0,755	13134565	87,95	33,69
	2	0,835	1578573	10,57	4,05
	3	0,859	221708	1,48	0,57
Lane 9	1	0,755	7859664	100,00	19,91
Lane 10	1	0,755	1678170	100,00	4,89
Lane 11	1	0,757	11775040	100,00	27,41
Lane 12	1	0,759	20960562	100,00	38,94
Lane 13	1	0,761	2350038	100,00	6,33
Lane 14	1	0,763	2520516	100,00	8,20
Lane 15	1	0,765	23319114	100,00	29,88
Lane 16	1	0,765	4054284	100,00	10,73
Lane 17	1	0,769	12695002	100,00	24,38
Lane 18	1	0,348	3504011	5,73	4,53
	2	0,398	8995847	14,70	11,62
	3	0,437	2349204	3,84	3,03
	4	0,457	2705107	4,42	3,49
	5	0,487	2844190	4,65	3,67
	6	0,519	2539273	4,15	3,28
	7	0,555	2356271	3,85	3,04
	8	0,596	8698885	14,22	11,24
	9	0,618	3286081	5,37	4,25
	10	0,646	2805932	4,59	3,62
	11	0,672	2411068	3,94	3,11
	12	0,704	1816034	2,97	2,35
	13	0,740	3559844	5,82	4,60
	14	0,777	8169637	13,35	10,55
	15	0,823	2250710	3,68	2,91
	16	0,867	56203	0,09	0,07
	17	0,917	2835902	4,64	3,66
Lane 19	1	0,767	13950189	100,00	24,67

Lane 20	1	0,761	3669036	100,00	8,89
Lane 21	1	0,761	13565280	100,00	26,27
Lane 22	1	0,759	25258332	100,00	44,75
Lane 23	1	0,757	14036920	100,00	29,66
Lane 24	1	0,755	22980885	100,00	51,62
Lane 25	1	0,753	25433055	100,00	47,69
Lane 26	1	0,749	30391380	100,00	47,72

[a] Band No., band number; [b] Relative Front, the relative movement of a band from top to bottom; [c] Volume, the sum of all intensities within the band; [d] Band %, percentage of a band volume compared to the volume of all bands in the lane; [e] Lane %, percentage of the band volume compared to the entire lane volume.

Table S1D. Densitometric analysis of the PCR-RFLP products obtained for *CXCR2* rs1126580 genotyping, shown in Figure S1D.

	Band No. [a]	Relative Front [b]	Volume [c] (Intensity)	Band % [d]	Lane % [e]
Lane 1	1	0,697	8215872	100,00	61,09
Lane 2	1	0,760	14089278	59,65	42,05
	2	0,831	9528918	40,35	28,44
Lane 3	1	0,760	5870802	42,35	19,12
	2	0,831	7991172	57,65	26,03
Lane 4	1	0,707	2742818	100,00	12,58
Lane 5	1	0,704	3192629	91,82	23,16
	2	0,763	284499	8,18	2,06
Lane 6	1	0,710	5010880	78,78	35,40
	2	0,765	1349720	21,22	9,53
Lane 7	1	0,707	3225183	85,20	22,62
	2	0,760	560060	14,80	3,93
Lane 8	1	0,712	3220956	80,78	16,78
	2	0,763	766574	19,22	3,99
Lane 9	1	0,704	4999048	100,00	33,13
Lane 10	1	0,702	6606289	81,88	43,47
	2	0,757	1461609	18,12	9,62
Lane 11	1	0,702	7436280	82,19	47,41
	2	0,755	1610880	17,81	10,27
Lane 12	1	0,707	978320	100,00	9,05
Lane 13	1	0,757	7779920	60,21	33,05
	2	0,821	5142120	39,79	21,84
Lane 14	1	0,694	4726400	100,00	30,33
Lane 15	1	0,697	16481298	51,24	37,79
	2	0,755	7148191	22,22	16,39
	3	0,823	8536403	26,54	19,57
Lane 16	1	0,749	16039774	72,03	44,79
	2	0,823	6229417	27,97	17,39
Lane 17	1	0,166	5621264	7,13	6,94
	2	0,227	15014692	19,06	18,55
	3	0,277	5892084	7,48	7,28
	4	0,301	4402068	5,59	5,44
	5	0,335	3252700	4,13	4,02
	6	0,372	2001032	2,54	2,47
	7	0,417	1375088	1,75	1,70
	8	0,464	11700392	14,85	14,46
	9	0,491	2522256	3,20	3,12
	10	0,522	2037024	2,59	2,52
	11	0,551	1014112	1,29	1,25

	12	0,588	685828	0,87	0,85
	13	0,631	3297932	4,19	4,07
	14	0,668	11812460	14,99	14,59
	15	0,715	2471788	3,14	3,05
	16	0,781	1670108	2,12	2,06
	17	0,826	4018828	5,10	4,97
Lane 18	1	0,739	8381242	64,60	32,40
	2	0,807	4592072	35,40	17,75
Lane 19	1	0,673	8457150	100,00	47,92
Lane 20	1	0,670	4862670	68,45	31,49
	2	0,731	616018	8,67	3,99
	3	0,810	1625488	22,88	10,53
Lane 21	1	0,665	5397773	47,34	22,05
	2	0,723	999416	8,77	4,08
	3	0,797	5004091	43,89	20,44
Lane 22	1	0,652	2301432	52,89	22,49
	2	0,726	122816	2,82	1,20
	3	0,799	1927094	44,29	18,84
Lane 23	1	0,710	5344840	59,95	25,85
	2	0,784	3570160	40,05	17,27

[a] Band No., band number; [b] Relative Front, the relative movement of a band from top to bottom; [c] Volume, the sum of all intensities within the band; [d] Band %, percentage of a band volume compared to the volume of all bands in the lane; [e] Lane %, percentage of the band volume compared to the entire lane volume.

Table S2. Distribution of the genotypes and alleles of *CCL2*, *CCL5* and *CXCR2* polymorphisms among the grades and stages of endometrial cancer.

Polymorphism	Genotype / Allele	No. [a] of genotypes / alleles (%)			*P*-value [b]	No. of genotypes / alleles (%)			*P*-value
		Grade				Stage			
		G1	G2	G3		I	II	III	
CCL2	C/C	5 (45.4%)	4 (36.4%)	2 (18.2%)		7 (63.6%)	1 (9.1%)	3 (27.3%)	
**903 T>C**	T/C	1 (100.0%)	0 (0.0%)	0 (0.0%)	0.787	2 (100.0%)	0 (0.0%)	0 (0.0%)	0.644
	T/T	50 (53.2%)	35 (37.2%)	9 (9.6%)		68 (73.1%)	13 (14.0%)	12 (12.9%)	
	Total	56 (52.8%)	39 (36.8%)	11 (10.4%)		77 (72.6%)	14 (13.2%)	15 (14.2%)	
	C	11 (47.8%)	8 (34.8%)	4 (17.4%)	0.503	16 (66.7%)	2 (8.3%)	6 (25.0%)	0.238
	T	101 (53.44%)	70 (37.04%)	18 (9.52%)		138 (73.4%)	26 (13.8%)	24 (12.8%)	
CCL5	A/A	6 (66.7%)	3 (33.3%)	0 (0.0%)		8 (80.0%)	1 (10.0%)	1 (10.0%)	
**-403 G>A**	G/A	12 (57.1%)	6 (28.6%)	3 (14.3%)	0.635	17 (80.9%)	3 (14.3%)	1 (4.8%)	0.421
	G/G	27 (51.9%)	21 (40.4%)	4 (7.7%)		32 (61.54%)	10 (19.23%)	10 (19.23%)	
	Total	45 (54.9%)	30 (36.6%)	7 (8.5%)		57 (68.7%)	14 (16.9%)	12 (14.4%)	
	A	24 (61.5%)	12 (30.8%)	3 (7.7%)	0.628	33 (80.5%)	5 (12.2%)	3 (7.3%)	0.155
	G	66 (52.8%)	48 (38.4%)	11 (8.8%)		81 (64.8%)	23 (18.4%)	21 (16.8%)	
**351 A>G**	A/A	43 (51.8%)	28 (33.7%)	12 (14.5%)		57 (70.4%)	11 (13.6%)	13 (16.0%)	
	A/G	7 (50.0%)	5 (35.7%)	2 (14.3%)	0.750	12 (85.7%)	2 (14.3%)	0 (0.0%)	0.552
	G/G	0 (0.0%)	1 (100.0%)	0 (0.0%)		1 (100.0%)	0 (0.0%)	0 (0.0%)	
	Total	50 (51.0%)	34 (34.7%)	14 (14.3%)		70 (72.92%)	13 (13.54%)	13 (13.54%)	
	A	93 (51.7%)	61 (33.9%)	26 (14.4%)	0.729	126 (71.6%)	24 (13.6%)	26 (14.8%)	0.236
	G	7 (43.75%)	7 (43.75%)	2 (12.5%)		14 (87.5%)	2 (12.5%)	0 (0.0%)	
CXCR2	A/A	27 (57.4%)	18 (38.3%)	2 (4.3%)	0.218	36 (78.3%)	3 (6.5%)	7 (15.2%)	0.206

**1440 G>A**	G/A	14 (46.7%)	10 (33.3%)	6 (20.0%)		17 (60.7%)	6 (21.4%)	5 (17.9%)	
	G/G	9 (47.4%)	6 (31.6%)	4 (21.0%)		12 (63.2%)	5 (26.3%)	2 (10.5%)	
	Total	50 (52.1%)	34 (35.4%)	12 (12.5%)		65 (69.9%)	14 (15.05%)	14 (15.05%)	
	A	68 (54.8%)	46 (37.1%)	10 (8.1%)	0.043	89 (74.2%)	12 (10.0%)	19 (15.8%)	0.034
	G	32 (47.05%)	22 (32.35%)	14 (20.6%)		41 (62.12%)	16 (24.24%)	9 (13.64%)	

[a] No., number; [b] Pearson's Chi-squared test; $P \leq 0.050$ is considered significant
